# Functional characterization of the type I toxin Lpt from *Lactobacillus rhamnosus* by fluorescence and atomic force microscopy

**DOI:** 10.1038/s41598-019-51523-z

**Published:** 2019-10-23

**Authors:** Stefano Maggi, Korotoum Yabre, Alberto Ferrari, Camilla Lazzi, Mitsuoki Kawano, Claudio Rivetti, Claudia Folli

**Affiliations:** 1 Department of Chemistry, Life Sciences and Environmental Sustainability, University of Parma, 43124 Parma, Italy; 2 Department of Food and Drug, University of Parma, 43124 Parma, Italy; 3 Department of Human Nutrition, Faculty of Contemporary Life Science, Chugokugakuen University, Niwase 83, Kita-ku, Okayama 701-0197 Japan

**Keywords:** Cellular microbiology, Fluorescence imaging, Atomic force microscopy

## Abstract

Lpt is a 29 amino acid long type I toxin identified in the plasmid DNA of wild *Lactobacillus rhamnosus* strains isolated from food. We previously reported that transcription of the encoding gene was upregulated under nutritional starvation conditions mimicking cheese ripening environment. The heterologous expression of the Lpt peptide in *E. coli* resulted in cell growth inhibition, nucleoid condensation and compromised integrity of the cell membrane. Fusion of the Lpt peptide with the fluorescent protein mCherry allowed to visualize the accumulation of the peptide into the membrane, while mutagenesis experiments showed that either the insertion of a negatively charged amino acid into the hydrophobic α-helix or deletion of the hydrophilic C-terminal region, leads to a non-toxic peptide. AFM imaging of Lpt expressing *E. coli* cells has revealed the presence of surface defects that are compatible with the loss of portions of the outer membrane bilayer. This observation provides support for the so-called “carpet” model, by which the Lpt peptide is supposed to destabilize the phospholipid packing through a detergent-like mechanism leading to the removal of small patches of bilayer through micellization.

## Introduction

Toxin-antitoxin (TA) systems are composed of a stable toxin, a protein or a peptide capable of targeting an essential cellular function, and an unstable antitoxin, a protein or a non-coding RNA able to counteract toxin activity. TA systems are widely distributed in plasmids and chromosomes of bacteria and archaea and are classified into six different types based on the nature and the mechanism of action of their antitoxins^1–3^. Type I TA systems are characterized by a hydrophobic toxin peptide and an RNA antitoxin able to interfere with the toxin peptide synthesis by interacting with its encoding mRNA. Nevertheless, in different type I TA systems, the RNA antitoxin alone is not sufficient to tightly block toxin expression and the translation of the toxin mRNA can occur only after processing^4–6^. Different physiological roles, such as plasmid maintenance^3^, stress-response^7^ and antibiotic resistance^8^, have been proposed for type I toxins together with different mechanisms of action. For instance, cytoplasmic toxins, such as SymE and RalR, catalyse nucleic acid cleavage, while membrane-associated toxins, such as Hok, Fst and LdrD, induce pore formation and/or nucleoid condensation^9–11^. *Hok/sok* from *E. coli* and *fst-RNAI/RNAII* from *E. faecalis* are among the best characterized type I TA systems. *Hok/sok* has been initially identified as a stabilization locus of the R1 plasmid^12^ but later studies have found several homologous systems in the chromosomal DNA of Gram-negative bacteria^13,14^, suggesting, for this TA system, roles other than post-segregational killing. Phase-contrast microscopy analysis of *E. coli* cells expressing the Hok toxin has revealed an unusual morphology, characterized by dense structures located at the cell poles^12,15^. Recently, it has been shown that the homologous HokB peptide, fused to the fluorescent protein mCherry, retains toxicity and is found to be associated with the cell membrane. *In vitro* experiments conducted using synthetic and natural lipid bilayers have demonstrated the capacity of the HokB peptide to form pores^8^.

The *Fst-RNAI/RNAII* TA system was initially identified in the pAD1 plasmid from *Enterococcus faecalis*^16^, however, later studies have found several homologous systems in the chromosome and plasmids of different Gram-positive bacteria^17,18^. Fluorescence microscopy studies carried out on *Enterococcus faecalis* and *Bacillus subtilis* have shown that the expression of the Fst peptide induces nucleoid condensation, cell division abnormalities and membrane damages^19,20^. Similar results have also been observed in the transmission electron micrographs of *Staphylococcus aureus* expressing a type I toxin peptide homologous to Fst^18^. NMR structural determination shows that Fst forms a membrane-binding α-helix in the N-terminal region with an intrinsically disordered and charged C-terminal tail pointing into the cytosol^21^.

Recently, a type I TA system, with a genetic architecture similar to that of the *fst-RNAI-RNAII* system, has been found in the plasmid DNA of *Lactobacillus rhamnosus*^22^, a non-starter lactic acid bacteria capable of adapting to adverse environmental conditions typical of long-ripened cheese, thus playing an important role in flavour development. The toxin encoding mRNA has been initially identified by transcriptomic experiments aimed at analysing sequences overexpressed by *L. rhamnosus* strains grown under conditions of nutrient starvation. Bioinformatics analyses of the putative TA *locus* has identified two convergently transcribed RNAs: RNAI encoding for a 29 amino acid toxin named Lpt, and RNAII, which is a non-coding RNA acting as an antitoxin. In this work, we report the functional characterization of the Lpt toxin in the heterologous *E. coli* C41(DE3) pLysS strain using fluorescence and atomic force microscopy analysis. The results show that the Lpt peptide inhibits *E. coli* growth and induces nucleoid compaction with the loss of membrane integrity. Membrane damage was directly visualized by AFM imaging of the cell surface, providing support for the destabilization of the phospholipid bilayer through a detergent-like mechanism of action.

## Results

### Lpt expression leads to growth inhibition, nucleoid condensation and membrane damage

**Figure 1:**
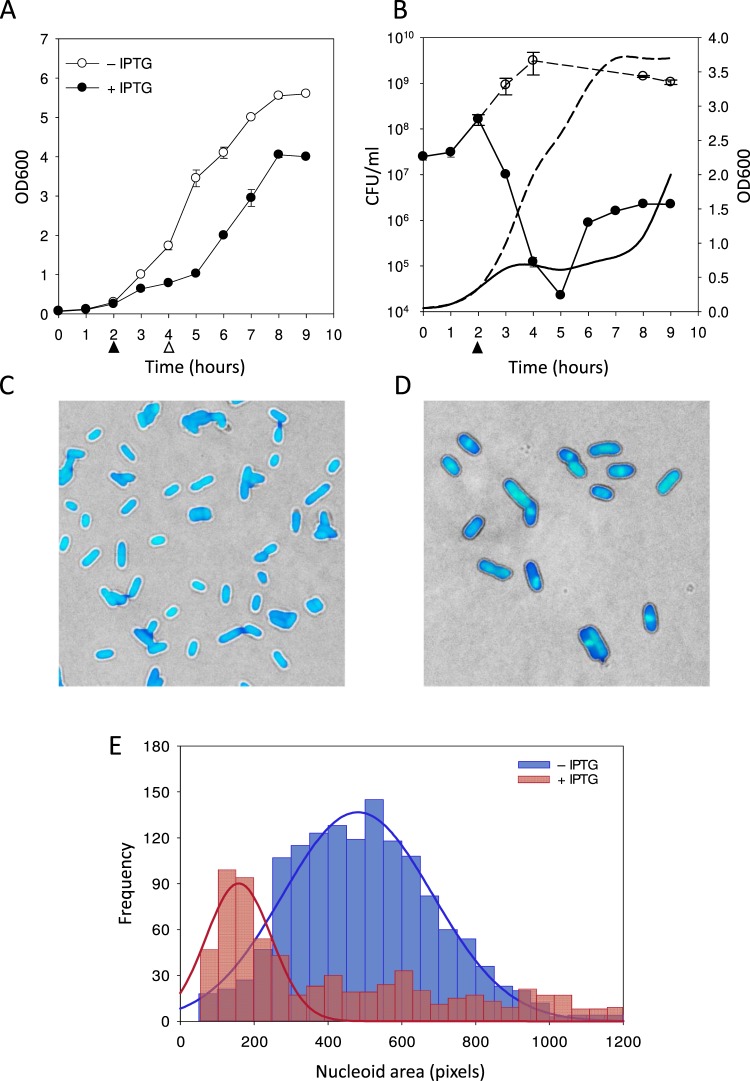
Effect of Lpt induction on *E. coli* growth and nucleoid morphology. (**A**) Growth curves of the recombinant *E. coli* C41(DE3) pLysS strain harbouring an inducible Lpt in the absence (open circles) and in the presence of IPTG (closed circles). Filled and empty arrows indicate the time points of IPTG addition and cell harvesting, respectively. Each data point represents the mean value ± SD of three independent experiments. (**B**) Plate counts of the recombinant *E. coli* C41(DE3) pLysS strain harbouring an inducible Lpt over time in the absence (open circles) and in the presence of IPTG (closed circles). Each data point represents the mean CFU/ml value ± SD of three different plates. Filled arrow indicates the time point of IPTG addition. Line plots represent the OD600 of induced (solid line) and non-induced (dashed line) cell growth. Representative bright-field/fluorescence overlaid images of non-induced (**C**) and two-hour induced (**D**) DAPI stained *E. coli* cells. (**E**) Distribution of nucleoid areas measured from the DAPI fluorescence images of non-induced (blue bars) and induced (red bars) *E. coli* cells. Distributions are fitted with a Gaussian function with the following mean ± SD parameters: blue curve mean 478 ± 203 pixels (N = 1380); red curve mean 153 ± 109 pixels (N = 703). The statistical significance of the difference between the mean of the two distributions was assessed by Student’s t-test with p ≤ 0.05.

In a previous study^22^, we analyzed the toxicity of the Lpt peptide in *E. coli* DH10bT1R transformed with the recombinant vector pSRKKm-lpt harbouring the Lpt coding sequence under the control of the lactose inducible *lac* promoter. Although in the presence of lactose, we observed growth inhibition, it was not possible to completely repress the promoter even in the presence of glucose. To circumvent this problem and to achieve a more stringent control of Lpt expression, in the present study, the Lpt coding sequence was cloned in the pET11b vector (pET11b-Lpt) which was then used to transform *E. coli* C41(DE3) pLysS cells. In the absence of IPTG, the growth curves of *E. coli* cells, transformed either with the empty pET11b or with pET11b-Lpt vectors, are highly similar (Supplementary Fig. S1), indicating complete repression of the toxic peptide. Lpt toxicity was verified by monitoring cell growth over time in LB liquid medium in the presence or in the absence of the inducer IPTG. As shown in Fig. 1A,B, the growth of *E. coli* cells is significantly inhibited after induction, confirming the toxic activity of the Lpt peptide. However, after three hours from induction, cell growth starts again as shown by the increased values of OD600 and CFU/ml, an effect also observed with other type I toxins^23^. To characterize the cell morphology during growth inhibition, we employed fluorescence microscopy and the membrane-permeable blue fluorescent dye 4′,6-diamidino-2-phenylindole (DAPI), which preferentially stains the nucleoid DNA. For this analysis, induced cells were harvested after two hours of induction and compared with non-induced cells grown for the same time. Figure 1C depicts non-induced cells showing an elongated nucleoid that occupies the entire cell volume. Conversely, with induced cells the DAPI fluorescence signal reveals a circular and more compact nucleoid located in the middle of the cell (Fig. 1D). To quantitatively analyse the observed nucleoid compaction, the area of the blue-stained nucleoid was measured (see Methods). As shown in Fig. 1E, the mean of the distribution of the nucleoid area shifts from 478 ± 203 pixels to 153 ± 109 pixels upon IPTG induction. This result confirms that the bacterial nucleoid undergoes condensation because of Lpt expression.

**Figure 2:**
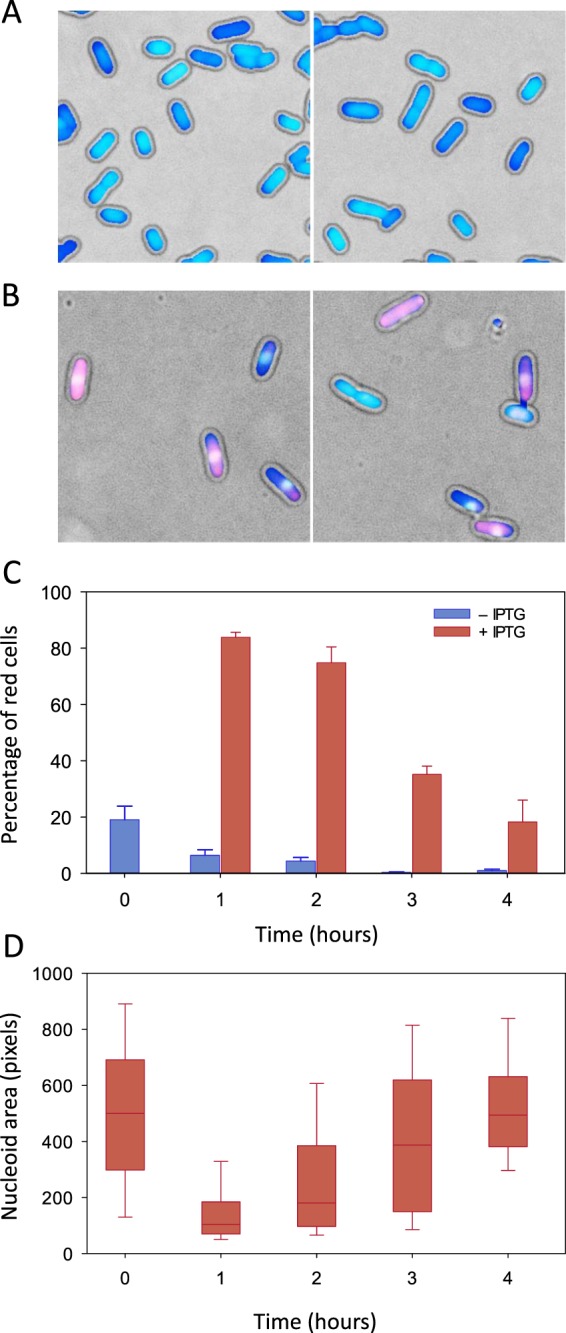
Evaluation of membrane integrity and nucleoid condensation by DAPI/EtBr staining. (**A**) Representative bright-field/fluorescence overlaid images of non-induced (**A**) and induced (**B**) *E. coli* cells stained with DAPI and Ethidium bromide dyes after two hours of induction. (**C**) Percentage of *E. coli* cells displaying the red EtBr fluorescence signal at different time points of growth after induction. Cells displaying both the blue and red fluorescence signal are considered red because of the loss of membrane integrity. Blue bars non-induced cells; red bars induced cells. Error bars represent the SD of three independent experiments. For the non-induced culture, the number of scored cells was: 2392 (t0), 2894 (1h), 3851 (2h), 5185 (3h) and 4433 (4h). For the induced culture, the number of scored cells was: 2562 (1h), 3741 (2h), 3987 (3h) and 1590 (4h). (**D**) Box-plot of the nucleoid area measured from fluorescence images of non-induced (t0) and induced *E. coli* cells at different time points of growth after induction (t1, t2, t3 and t4). The boundaries of the box plot indicate the 25th and 75th percentile of the distribution, the horizontal line within the box represents the median and the error bars show the 10th and 90th percentile of the distribution. The total number of cells represented in each box-plot from t0 to t4 is: 457, 1017, 762, 509, 442. The Kruskal-Wallis H test indicates that there is a statistically significant difference among data distributions with the exception of t0 and t4 for which P > 0.05.

It is known that nucleoid condensation caused by some type I toxins is often associated with the loss of membrane integrity^19,20,24^. To assess whether Lpt expression affects membrane integrity, we performed a set of experiments using DAPI and ethidium bromide (EtBr) staining. This protocol combines the membrane-permeable fluorescent dye DAPI, which stains all bacteria, with the membrane-impermeable fluorescent dye EtBr, which permeates only de-energized bacteria^25^ or bacteria with damaged membranes^26^. *E. coli* C41(DE3) pLysS cells carrying pET11b-Lpt were grown in liquid media for two hours (t_0_) and were induced by the addition of 1 mM IPTG. Aliquots of non-induced and induced cells were collected for the following four hours, stained with DAPI/EtBr and analysed by fluorescence microscopy as described in Methods (Fig. 2 and Supplementary Images S1–S5). Cells showing some degree of red fluorescence intensity are considered red, i.e. with the loss of membrane integrity. As shown in Fig. 2C, at time t_0_, 19 ± 5% of the cells are red while the remaining 81% are blue. The number of red cells decreases rapidly in the non-induced fraction, reaching values close to zero after three hours. Conversely, in the case of induced cells, the fraction of red cell increases up to 84 ± 2% during the first hour, then decreasing to 18 ± 8% during the following three hours.

By employing the blue fluorescence signal of DAPI, the same set of images was also used to quantify the degree of nucleoid condensation at each time point. As shown in Fig. 2D, during the first hour, the nucleoid area undergoes a drastic reduction, then returning to a normal size in the following three hours.

These results strongly suggest that Lpt expression affects membrane integrity and induces nucleoid condensation. However, in agreement with the growth curve behaviour, after three hours from induction, most *E. coli* cells recover from the toxin-induced membrane damage, and the nucleoid regains a normal uncondensed state.

### *In vivo* localization of Lpt-mCherry fusion protein

To investigate Lpt localization within the bacterial cell, the red fluorescent protein mCherry was fused to the C-terminus of the toxic peptide. The rationale of this strategy is that, based on Fst similarity^22^, mCherry fusion at the more hydrophilic and probably cytosolic C-terminal domain may have little impact on membrane localization. To minimize the interference with Lpt folding, a linker of eight amino acids (GGGSGGGS) was inserted between the two polypeptides.

**Figure 3:**
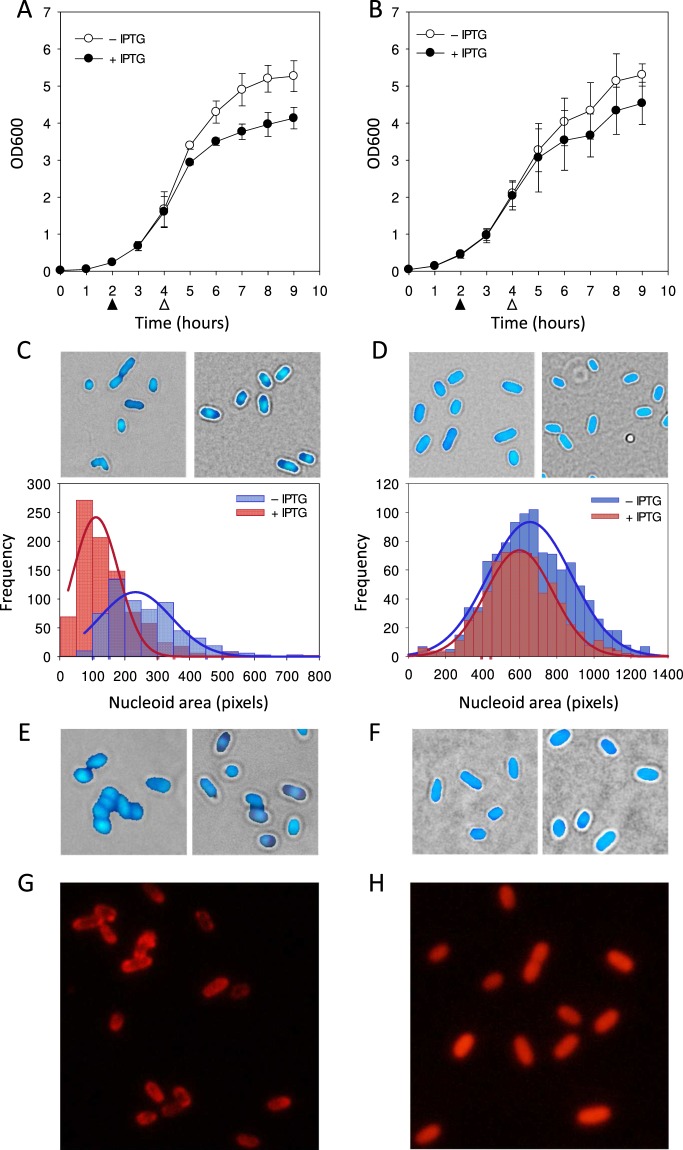
Effect of Lpt-mCherry induction on *E. coli* growth, nucleoid morphology and membrane integrity. Growth curves of the recombinant *E. coli* C41(DE3) pLysS strain harbouring an inducible Lpt-mCherry (**A**) or mCherry (**B**) in the absence (open circles) and in the presence of IPTG (closed circles). Filled and empty arrows indicate the time points of IPTG addition and cell harvesting, respectively. Each data point represents the mean value ± SD of three independent experiments. (**C**) Representative bright-field/fluorescence overlaid images of non-induced (left panel) and two-hour induced (right panel) *E. coli* cells expressing Lpt-mCherry and stained with DAPI. Distribution of nucleoid areas measured from the DAPI fluorescence images of non-induced (blue bars) and induced (red bars) *E. coli* cells. Distributions are fitted with a Gaussian function with the following mean ± SD parameters: blue curve mean 233 ± 111 pixels (N = 612); red curve mean 111 ± 70 pixels (N = 902). (**D**) Representative bright-field/fluorescence overlaid images of non-induced (left panel) and two-hour induced (right panel) *E. coli* cells expressing mCherry protein and stained with DAPI. Distribution of nucleoid areas measured from the DAPI fluorescence images of non-induced (blue bars) and induced (red bars) *E. coli* cells. Distributions are fitted with a Gaussian function with the following mean ± SD parameters: blue curve mean 654 ± 225 pixels (N = 1051); red curve mean 598 ± 182 pixels (N = 684). (**E**) Representative bright-field/fluorescence overlaid images of non-induced (left panel) and two-hour induced (right panel) *E. coli* cells expressing Lpt-mCherry and stained with DAPI/EtBr. (**F**) Representative bright-field/fluorescence overlaid images of non-induced (left panel) and two-hour induced (right panel) *E. coli* cells expressing mCherry protein and stained with DAPI/EtBr. (**G**) mCherry fluorescence signal of *E. coli* cells expressing the fused Lpt-mCherry protein. (**H**) mCherry fluorescence signal of *E. coli* cells expressing the mCherry protein. The statistical significance of the difference between the mean of the two distributions in C e D was assessed by Student’s t-test with p ≤ 0.05.

Growth assays of *E. coli* transformed with the inducible expression vector pET11b-Lpt-mCherry were carried out to assess the toxicity of the fused protein. As shown in Fig. 3A, the expression of Lpt-mCherry affects cell growth; however, compared to Lpt-expressing cells (Fig. 1A), inhibition is delayed by about three hours and the global inhibitory effect is lower. In a control experiment carried out with *E. coli* expressing the fluorescent mCherry protein alone, no significant growth inhibition was observed (Fig. 3B).

To assess membrane integrity, nucleoid condensation and cellular localization of Lpt-mCherry, a DAPI/EtBr fluorescence microscopy analysis, combined with the analysis of the Lpt-mCherry red fluorescent signal, was performed. Non-induced and induced *E. coli* cells harvested after 2 hours from induction and stained with DAPI were imaged by fluorescence microscopy (Fig. 3C). The intense blue fluorescent spot visible at the centre of induced cells indicates nucleoid condensation. The quantitative analysis shown by histograms in Fig. 3C confirms that, upon induction, the mean of the nucleoid area distribution shifts to lower values. The same analysis performed with *E. coli* expressing mCherry alone shows no evidence of nucleoid condensation (Fig. 3D).

Induced and non-induced samples were also stained with DAPI/EtBr to verify membrane integrity. As shown in Fig. 3E, induced cells display mainly the blue DAPI signal together with a weak red signal of EtBr, suggesting that Lpt-mCherry affects membrane integrity but with a lower extent compared to wt Lpt. Again, the expression of mCherry protein alone does not affect membrane permeability (Fig. 3F).

The same cell samples were also analysed without staining to detect mCherry fluorescence. As shown in Fig. 3G,H, the red fluorescence signal of Lpt-mCherry appears more intense in the proximity of the cell membrane (Fig. 3G), whereas the fluorescence signal of mCherry alone is uniformly diffused as expected for a cytosolic protein (Fig. 3H). These results support the hypothesis that Lpt localizes into the membrane, affects membrane permeability and causes nucleoid condensation.

### Changing the hydrophobic/hydrophilic pattern affects Lpt toxicity

**Figure 4:**
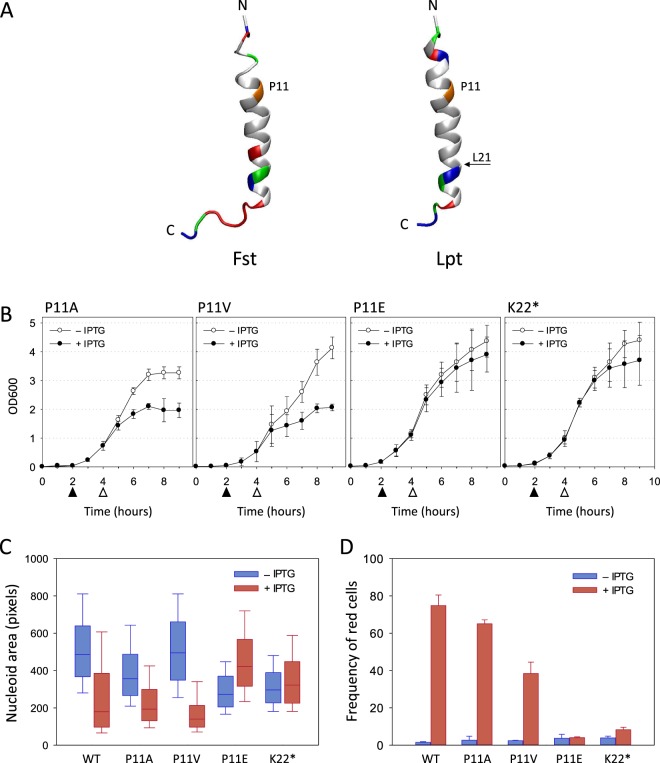
Growth curves, nucleoid morphology and membrane integrity assays of *E. coli* cells expressing Lpt mutants. (**A**) NMR structure of Fst peptide (PDB ID: 2vk5) (left) and structural homology model of *L. rhamnosus* Lpt peptide (right) build with the Swiss-PdbViewer software^41^ by using the Fst structure as template. Structural illustration was generated using VMD software^42^. Residue colour code: light grey, hydrophobic; red, negatively charged; blue, positively charged; green, uncharged hydrophilic; orange, proline 11. (**B**) Growth curves of the recombinant *E. coli* C41(DE3) pLysS strain harbouring an inducible Lpt mutant in the absence (open circles) and in the presence of IPTG (closed circles). Filled and empty arrows indicate the time points of IPTG addition and cell harvesting, respectively. Each data point represents the mean value ± SD of three independent experiments. (**C**) Box-plot of nucleoid areas measured from the DAPI fluorescence images (Supplementary Figs S2 and S3) of non-induced (blue bars) and induced (red bars) *E. coli* cells expressing Lpt wild-type and mutants. The boundaries of the box plot indicate the 25th and 75th percentile of the distribution, the horizontal line within the box represents the median and the error bars show the 10th and 90th percentile of the distribution. The total number of cells in each box-plot from left to right is: 685, 762, 1056, 787, 601, 1164, 478, 619, 1136, 973. Each pair of box-plots was subjected to the Mann–Whitney U test to verify the statistical significance of the difference between the two distributions. p ≤ 0.001 for all cases. (**D**) Percentage of non-induced (blue bars) and induced (red bars) *E. coli* cells displaying the red EtBr fluorescence signal after two hours of induction. Error bars represent the SD of three independent experiments. For each Lpt variant, from left to right, the number of scored cells was: 3926, 1833, 4317, 3863, 5342, 5210, 4105, 3411, 4715, 3077.

In a previous study regarding the *Enterococcus faecalis* Fst peptide, it was shown that a proline at position 11 is crucial for toxicity^18^. In particular, the substitution of Pro11 with either alanine, serine, glutamate or lysine does not cause growth arrest, and the mutated Fst peptides have therefore been classified as non-toxic. Likewise, the Lpt peptide carries a proline residue at position 11, which is highly conserved among all the Lpt homologs found in the plasmid DNA of lactic acid bacteria^22^. Therefore, it was of interest to investigate the effect of Pro11 substitution with amino acids affecting the hydrophobicity of the peptide. Although sequence homology between Fst and Lpt is low (30%), we built a structural homology model of Lpt using Fst coordinates^21^ as a template (Fig. 4A) with the aim of facilitating the comparison between Fst and Lpt residue-type distribution and to better comprehend the rationale behind the point mutations.

Three different Lpt mutants were obtained by replacing Pro11 with either alanine, valine or glutamic acid. As shown in Fig. 4B, upon induction the P11A and P11V mutants inhibit *E. coli* growth; however, compared to wild-type Lpt, growth inhibition is delayed by about three hours. Conversely, the P11E mutant does not inhibit cell growth, as shown by the highly similar growth curves of non-induced and induced cultures (Fig. 4B).

To evaluate the degree of nucleoid compaction, the P11A, P11V and P11E mutants have been analyzed by fluorescence microscopy using DAPI staining as described above for wt Lpt. As shown in Supplementary Figs S2 and S3 and summarized in Fig. 4C, the expression of P11A and P11V Lpt mutants induces nucleoid compaction with the median of the nucleoid area shifting from 356 pixels to 193 pixels and from 495 pixels to 140 pixels, respectively. Conversely, P11E does not cause nucleoid compaction in line with the absence of growth inhibition. In this particular case, an increase of the nucleoid area upon induction was observed, an effect probably due to the over-expression of a recombinant non-toxic peptide.

Lpt-induced membrane damage has been evaluated by analysing the number of red *E. coli* cells harvested after two hours of induction using DAPI/EtBr staining in a fluorescence microscopy experiment. Figure 4D shows the percentage of red cells in induced and non-induced samples, with respect to the total cells in the microscope view for each of the three Lpt mutants analyzed. A large number of red cells were observed for the P11A and P11V mutants (65.0 ± 2.1% and 38.4 ± 6.1%, respectively) whereas, for the P11E mutant the number of red cells was 4.0 ± 0.5%, a value very similar to that of the non-induced sample (3.6 ± 2.1%).

Overall, the results obtained with Lpt point mutants indicate that the substitution of proline 11 with the hydrophobic amino acids alanine or valine results in a toxic peptide, while the substitution of proline 11 with the hydrophilic glutamate leads to a non-toxic peptide. Based on these data, we propose that the P11A and P11V Lpt mutants retain the ability to localize into the membrane causing the loss of integrity. On the other hand, the charged amino acid introduced with the P11E mutation should prevent membrane localization; thus, preserving the membrane integrity. To further investigate this aspect, the P11E mutation was introduced into the Lpt-mCherry construct to monitor protein localization. *E. coli* C41(DE3) pLysS cells transformed with pET11b-LptP11E-mCherry display a standard growth curve (Supplementary Fig. S4A) and DAPI/EtBr fluorescence microscopy did not show any signs of membrane damage, nor nucleoid compaction (Supplementary Fig. S4B), confirming that the P11E mutation suppresses Lpt toxicity. Analysis of the mCherry fluorescence shows a uniformly diffuse red signal (Supplementary Fig. S4C), validating the hypothesis that the glutamate at position 11 prevents Lpt localization into the membrane.

NMR structural data of the Fst peptide^21^ have shown that the N-terminal alpha-helix represents the membrane-binding domain, whereas the intrinsically disordered charged region at the C-terminal is exposed into the cytosol. The deletion of eight amino acids from the charged C-terminal domain of the Fst peptide does not eliminate the toxicity^18^.

To evaluate a possible role of the Lpt C-terminal region, we introduced a stop codon after L21 in order to produce a truncated Lpt peptide lacking eight amino acids (KYALDNHK) at the C-terminus. As shown in Fig. 4, the truncated Lpt peptide is essentially non-toxic as indicated by the normal growth curve (Fig. 4B), by the absence of nucleoid condensation (Fig. 4C) and by the absence of membrane damage (Fig. 4D). Thus, differently from Fst, the hydrophilic C-terminal tail of Lpt, most probably exposed into the cytosol, is essential for toxicity.

### Atomic force microscopy visualization of Lpt induced membrane damages

**Figure 5:**
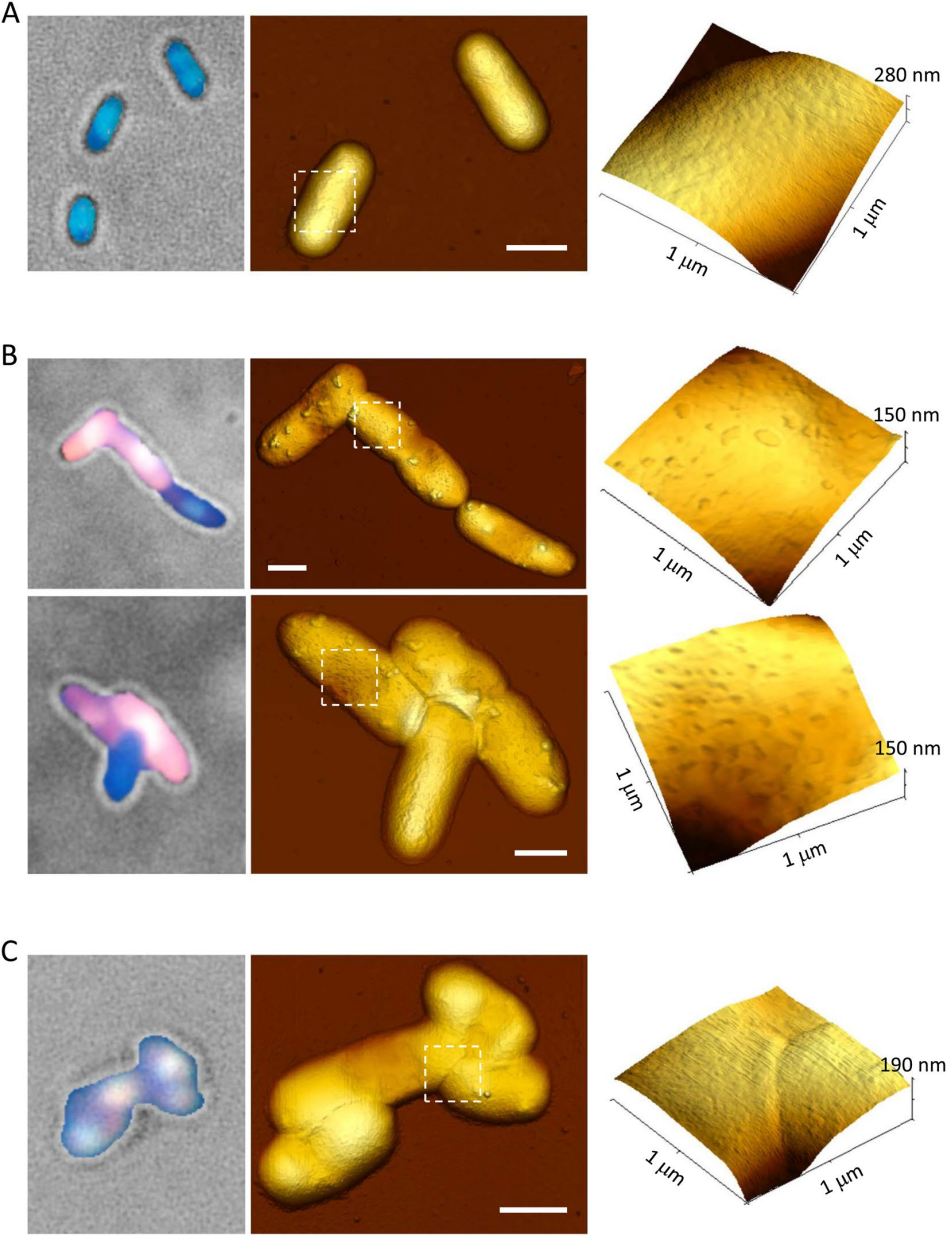
Atomic force microscopy analysis of *E. coli* cells expressing wt Lpt and Lpt-mCherry. Left column, bright-field/fluorescence overlaid images of *E. coli* cells stained with DAPI/EtBr after two hours of induction. Middle column, AFM top views of the same *E. coli* cells shown on the left. Size bar equals to 1 μm. Right column, 3D view of the cell surface details of the region delimited by the dashed white box. Recombinant *E. coli* C41(DE3) pLysS cells harbouring an inducible wt Lpt in the absence (**A**) or in the presence (**B**) of IPTG. (**C**) *E. coli* C41(DE3) pLysS cells expressing Lpt-mCherry in the presence of IPTG.

To have further insight into the loss of membrane integrity upon Lpt induction, we have employed atomic force microscopy (AFM) imaging to visualize the surface morphology of induced and non-induced *E. coli* cells. AFM is an ideal tool for this investigation because it provides high-resolution topographic images of the entire bacterial cell without the use of an external means of contrast. For the AFM analysis, *E. coli* cells were grown in LB liquid medium, harvested, washed with PBS and deposited onto glass as described in Methods. AFM imaging was performed in air with a Park XE-100 microscope (Park Systems) operating in intermittent mode. As shown in Fig. 5A and in Supplementary Fig. S5A, non-induced *E. coli* display the typical rod-shaped morphology with a height of about 250 nm, characteristic of swollen cells. A detailed inspection of the cell surface shows an intact structure with some degree of roughness but without any visible damage. Conversely, *E. coli* cells expressing wt Lpt display a similar rod-like shape but with height in the range of 100 to 200 nm, which indicates deflated cells (Supplementary Fig. S5B). The cell surface is characterized by the presence of large protruding globular features that may represent cell wall structures or cytoplasmic granules that becomes evident when the cell deflates (Fig. 5B). A more detailed inspection of the cell surface reveals the presence of small oblong-shaped depressions with a lateral dimension ranging from 20 to 150 nm and a depth of 6.2 ± 0.7 nm (3D view of Fig. 5B). These depressions of the membrane surface are characteristic of EtBr permeable cells (red cells) and have never been observed in non-induced cells or cells expressing non-toxic Lpt peptides (see below). In a control experiment, aimed at verifying that the observed membrane damage is characteristic of Lpt expressing cells, *E. coli* was treated with chloramphenicol which is known to cause nucleoid compaction and the loss of membrane integrity^27^. As shown in Supplementary Fig. S6, in the presence of chloramphenicol, DAPI/EtBr stained cells display, as expected, the red EtBr signal and a compacted toroidal nucleoid; however, no visible surface damage is revealed by AFM (3D view in Supplementary Fig. S6).

**Figure 6:**
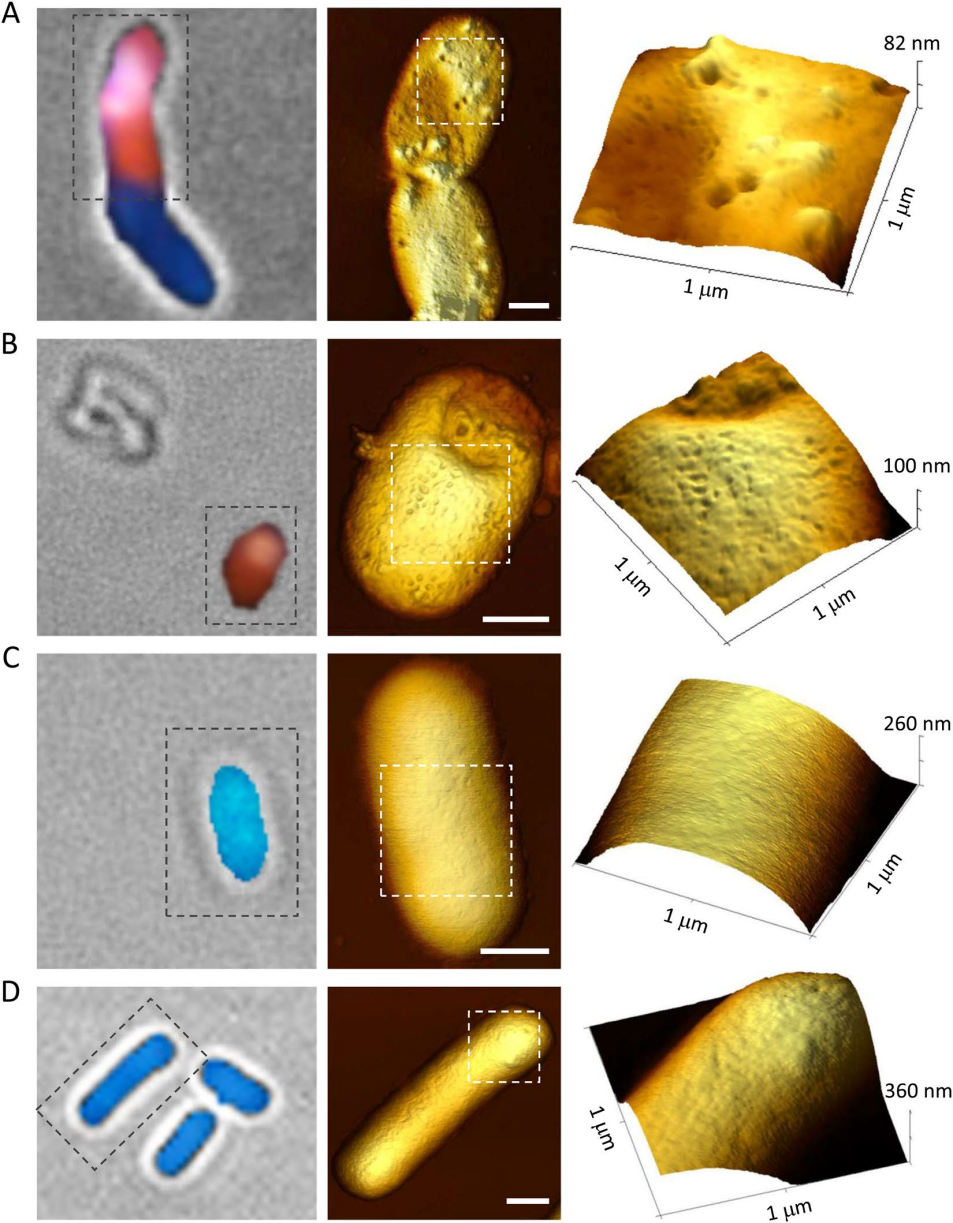
Atomic force microscopy analysis of *E. coli* cells expressing Lpt mutants. Left column, bright-field/fluorescence overlaid images of *E. coli* cells stained with DAPI/EtBr after two hours of induction. Middle column, AFM top views of the same *E. coli* cells shown on the left. Size bar equals to 0.5 μm. Right column, 3D view of the cell surface details of the region delimited by the dashed white box. (**A**) Lpt P11A mutant. (**B**) Lpt P11V mutant. (**C**) Lpt P11E mutant. (**D**) Truncated Lpt mutant.

The AFM surface characterization has also been performed on *E. coli* cells expressing the fusion protein Lpt-mCherry described above, or a mutated form of the Lpt peptide. In the case of Lpt-mCherry, the bacteria surface appears smooth and very similar to that observed for non-induced cells (Fig. 5C). Whereas, in the case of P11A and P11V mutants, cells display the same phenotype of wt Lpt, characterized by deflated cells, the presence of protruding globular features and round-shaped depressions (Fig. 6A,B). Conversely, *E. coli* cells expressing P11E or the truncated Lpt peptide are characterized by swollen cells without particular structural features or damages (Fig. 6C,D).

The results obtained by AFM indicate that the expression of a toxic Lpt peptide induces, on the surface of the bacteria, the formation of characteristic inward depressions which correlate well with the loss of membrane integrity. Only in the case of Lpt-mCherry, for which we observed a weak loss of membrane integrity (cells are not as red as wt Lpt), the surface damage was not perceivable by the AFM tip. These results also show that AFM can be a useful tool for providing visual inspection of the membrane damage caused by type I bacterial toxins.

## Discussion

The abundance of type I TA systems in bacterial genomes is likely underestimated due to the difficulty in the biochemical identification of small toxin peptides and in the bioinformatics prediction of short non-coding antitoxin RNAs. Type I TA systems carried by plasmid DNA are generally associated with plasmid stability and maintenance, but other biological roles have also been proposed^28^. Type I toxins are generally small hydrophobic peptides (less than 60 amino acids) containing a putative transmembrane α helix that can affect membrane integrity by forming pores or by acting as a detergent. In the case of pore-forming peptides, such as Hok or TisB toxins, the functional model is based on the capability of these toxins to interact with the cellular membrane where they can form pores responsible for reducing the transmembrane electrochemical gradients and the proton motive force, leading to cell growth arrest or cell lysis^9,29^. A second group of membrane-associated type I toxins, besides causing membrane stress, can also induce nucleoid condensation with a resulting “domino effect” leading to cell death or dormancy^9^. Among these toxins, the Fst peptide encoded by the pAD1 plasmid of *E. faecalis* is the best characterized. Its function is linked to a post-segregational killing mechanism leading to cell death upon the loss of pAD1 plasmid during cell division. Under these circumstances, the unstable antitoxin RNA is degraded allowing the Fst peptide to accumulate into the membrane and to induce nucleoid condensation, alteration of peptidoglycan synthesis and overexpression of membrane transporters^20,30^.

In a recent study, a type I toxin named Lpt was identified in the plasmid DNA of wild *L. rhamnosus* strains isolated from food sources. Transcription of the toxin encoding gene was upregulated under nutritional starvation conditions aimed to mimic the cheese ripening environment. In an initial attempt to study the activity of the Lpt toxin, the Lpt coding sequence was cloned into the pSRKKm plasmid under the control of the IPTG inducible *lac* promoter. Although this plasmid has been engineered to have a very low basal expression, it was not possible, even in the presence of glucose, to completely repress Lpt synthesis; thus, under not inducible conditions, some toxicity remained^22^. To circumvent this problem, in the present work, the Lpt coding sequence was cloned into a pET vector under the control of a T7 promoter and the construct was used to transform *E. coli* C41(DE3) pLysS cells, a strain expressing a small amount of T7 lysozyme which suppresses the basal expression of T7 RNA polymerase prior to induction. This system guarantees a tightly repressed expression of the recombinant protein under non-inducible conditions, and it is particularly suited for the expression of toxic proteins^31^. Although this recombinant system may produce an amount of toxin higher than that produced under physiological conditions, data in the literature have shown that the C41(DE3) pLysS *E. coli* strain has a mutation in the T7 promoter that significantly decreases the amount of T7 RNAP available and, in turn, the amount of recombinant protein synthetized. We further verified this conclusion by comparing the production of the non-toxic mCherry protein in C41(DE3) pLysS versus BL21 cells. The gel reported in Supplementary Fig. S7 confirms that after two hours of induction, C41(DE3) pLysS cells transformed with pET11b-mCherry produce at least ten times less mCherry protein than BL21 cells transformed with the same plasmid. These genetic features confer high resilience versus toxic proteins; thus, the C41(DE3) pLysS strain represents an obliged choice to study the *in vivo* activity of the Lpt peptide.

Induction of a functional Lpt in *E. coli* C41(DE3) pLysS causes growth arrest; however, after three hours, the cell growth starts again. This behaviour is probably due to the selection of resistant cells under the pressure of the toxic protein triggered by IPTG. Interestingly, similar growth assays conducted in *E. coli* BL21(DE3) were unsuccessful because of the impossibility to observe growth arrest upon Lpt induction (data not shown). In this latter case, resistant selection might have taken place even before IPTG induction, due to the not completely repressed basal expression of BL21(DE3) cells.

Beside growth arrest, expression of the Lpt peptide is also linked to other phenotypes characteristic of membrane-associated type I toxins, namely nucleoid condensation and the loss of membrane integrity, as demonstrated by fluorescence microscopy. Evidence favouring Lpt membrane localization comes from fluorescence microscopy experiments showing that the fused protein Lpt-mCherry accumulates into the membrane. The lower toxicity displayed by Lpt-mCherry is probably ascribable to the bulky mCherry fold attached at the Lpt C-terminus. Lpt membrane localization is further supported by point mutation experiments showing that the substitution of proline 11 with a negatively charged amino acid leads to a non-toxic peptide while the substitution of proline 11 with hydrophobic amino acids maintains toxicity. However, upon induction, P11A and P11V mutants display a delay in the growth inhibition and a lower number of red cells. This suggests that the proline-induced distortion of the α-helix is an important feature for the interaction with the lipid bilayer^32^ and thus has a role in toxicity.

Surface visualization of Lpt expressing *E. coli* cells carried out by AFM reveals the presence of inward patches with a lateral dimension varying from 20 to 150 nm and a depth of about 6 nm which corresponds to the thickness of the lipid bilayer. These membrane patches are probably formed by the disruption of the lipid bilayer as a consequence of the interaction with the Lpt peptide which may act through the so-called “carpet” mechanism that has been initially proposed to describe the mode of action of the antibacterial peptides dermaseptin^33^ and melittin^34^. According to this model, as the concentration of the Lpt peptides in the periplasmic space increases, Lpt forms a “carpet” across the inner surface of the outer membrane which destabilizes the phospholipid packing through a detergent-like mechanism leading to the removal of small portions of the bilayer through micellization^9,33^. However, we cannot exclude the formation of transmembrane channels by bundles of Lpt α-helical peptides as proposed by the barrel-stave mechanism^35,36^, because such pores would be too small to be revealed by the AFM tip under our experimental conditions. Nevertheless, we tend to disfavour this latter hypothesis because pore-forming peptides are characterized by secondary amphipathicity (*i.e*. hydrophilic/charged residues and hydrophobic residues exposed on opposite faces of the helix) which is not displayed by the Lpt peptide (Fig. 4A).

It should be considered that the phenomenon described here is occurring in a Gram-negative bacterium; therefore, in order to reach the outer membrane, the Lpt peptide has to diffuse through the inner membrane and the thin peptidoglycan layer. The inner membrane is probably permealized with a similar mechanism, while the peptidoglycan may be crossed in virtue of the small size of the Lpt peptide. Notably, α-helical antimicrobial peptides, with length and structure similar to type I toxins, are active also on Gram-positive bacteria coated with a thick layer of peptidoglycan^37,38^. In addition, the C41(DE3) pLysS *E. coli* strain produces a small amount of T7 lysozyme that may weaken the peptidoglycan wall^31^.

Interestingly, the fused protein Lpt-mCherry, which we have shown to be less toxic than wt Lpt and to determine membrane permeabilization to a lesser extent, does not form the inward membrane patches described above. We speculate that the large mCherry fold may reduce the diffusion towards the periplasmic space and also reduce the surfactant-like qualities of linked Lpt.

Further support to the detergent-like mechanism comes from the mutagenesis analysis of the Lpt peptide. In fact, by changing the amphipathic character of the peptide, namely through the insertion of a charged amino acid into the hydrophobic α-helical segment or the removal of the hydrophilic carboxy-terminal region, resulted in a non-toxic peptide.

In conclusion, we have demonstrated the toxicity of the recombinant Lpt peptide from *Lactobacillus rhamnosus* in the heterologous C41(DE3) pLysS *E. coli* strain. The use of this host was necessary because of the tight repression and low level of expression of the recombinant protein. Being Gram-negative, it also has the advantage to display an outer lipid membrane where the action of the toxic peptide can be directly monitored with the AFM. Overall, the data indicate that the Lpt peptide causes *E. coli* growth arrest with the loss of membrane integrity and nucleoid compaction, providing support for the destabilization of the phospholipid bilayer through a detergent-like mechanism of action.

## Methods

### Gene cloning, site-directed mutagenesis and *E. coli* transformation

*Lactobacillus rhamnosus* 1019 was isolated from Parmigiano Reggiano cheese as described in^39^. Bacteria were grown in Mann Rogosa Sharp medium (MRS) at 37 °C under anaerobiosis. Total DNA was isolated as described in^22^. The Lpt coding was amplified by PCR using *L. rhamnosus* 1019 total DNA, GoTaq DNA polymerase (Promega) and primers Lpt-plus/Lpt-minus (Supplementary Table S1). The amplicon was cloned into the pGEM-TEasy vector (Promega) and sub-cloned in the NdeI/BamHI restriction sites of the inducible expression vector pET11b (Novagen) to obtain the recombinant plasmid pET11b-Lpt (Supplementary Table S2).

Lpt peptide variants P11A (CCG>GCG), P11V (CCG>GTG), P11E (CCG>GAG) and truncated-Lpt (AAA>TAA) were obtained by site-directed mutagenesis using plasmid pET11b-Lpt as template, the high-fidelity PfuUltra II Fusion HS DNA polymerase (Stratagene) and mutagenic primers (Supplementary Table S1). Each reaction was treated with DpnI enzyme (NEB) and used to transform *E. coli* XL1-Blue cells. Mutations were verified by sequencing. Vectors pET11b-Lpt-mCherry and pET11b-mCherry, optimized for expression in *E. coli*, were custom made and purchased from Genscript. pET11b-LptP11E-mCherry was obtained by site-directed mutagenesis as described above using plasmid pET11b-Lpt-mCherry as template. All recombinant vectors (Supplementary Table S2) were used to transform *E. coli* C41(DE3) pLysS cells (Lucigen) by electroporation.

### Growth assays

The toxic activity of wild type, mutated and chimeric Lpt peptides was evaluated by analysing the growth of *E. coli* C41(DE3) pLysS cells transformed with the inducible vector pET11b harbouring the corresponding coding sequences. Single colony cell cultures were grown in LB liquid medium. The cultures were incubated at 37 °C for 9 hours and, at time intervals of one hour, the OD600 was measured with the spectrophotometer. After two hours of growth, the cultures were split into two equal aliquots, one aliquot was induced by the addition of 1 mM IPTG while the other was kept unaltered (non-induced). In the case of wild type Lpt, cell growth was also monitored by CFU/ml counting. At each time point, 1 ml of cell cultured was used to perform decimal dilutions in LB medium, followed by plating on LB-agar and incubation at 37 °C overnight. Colony counting was performed by using OpenCFU software^40^ with images captured by ChemiDoc MP Imaging System (Bio-Rad).

### Fluorescence and atomic force microscopy

*E. coli* samples used for fluorescence microscopy analysis were prepared starting from single colonies of freshly transformed *E. coli* C41(DE3) pLysS cells grown overnight on solid media. After two hours of growth in liquid medium (t_0_), the culture was split into two fractions: one was kept non-induced while the other was induced by the addition of 1 mM IPTG. Non-induced and induced cells were collected every hour for the following four hours. Harvested cells were washed three times with 1 ml of PBS and re-suspended in 50 μl of PBS for staining. DAPI and Ethidium bromide dyes were added to a final concentration of 10 μg/ml each, followed by 5-minute incubation at RT, washed twice with 1 ml of PBS to remove the excess of dye, resuspended in 50 μl of PBS and imaged immediately. The glass coverslips were functionalized with poly-L ornithine (0.01%, Sigma) to favour cell adhesion. An aliquot of 25 μl of the PBS cell suspension was deposited onto the coverslip for 30 seconds, washed with Milli-Q water and dried by evaporation at RT. Fluorescence images were taken with a Nikon Eclipse E600 microscope equipped with a 100X oil immersion objective and with a Nikon DS-Fi2 digital camera. For DAPI/EtBr staining the UV-2A filter was used. For mCherry expressing cells the Texas Red filter was used. Cells were focused in bright-field and images were recorded with an exposure time of 800 msec. Quantification of fluorescence images was performed using Matlab (see Supplementary Material). Histograms and boxplots were built with Sigmaplot (Systat Software, Inc.). The difference between the distributions was tested using Student’s t-test or Mann-Whitney U test as indicated in the figure legends.

AFM imaging was performed on the dried samples with a Park XE-100 microscope (Park Systems) operating in intermittent mode with a scan rate of 0.5 Hz. Commercial diving board silicon cantilevers (MikroMasch) were used. Images were processed with the Gwyddion software.

## Supplementary information

Supplementary Information
