# Supplementary material for: Functional characterization of the type I toxin Lpt from Lactobacillus rhamnosus by fluorescence and atomic force microscopy
Source: Sci Rep. 2019 Oct 23;9:15208. doi: 10.1038/s41598-019-51523-z (PMC6811638; doi:10.1038/s41598-019-51523-z)
Supplement: Supplementary file 1 — Supplementary Information [file 41598_2019_51523_MOESM1_ESM.pdf]

Functional characterization of the type I toxin Lpt from *Lactobacillus rhamnosus* by fluorescence and atomic force microscopy

Stefano Maggi, Korotoum Yabre, Alberto Ferrari, Camilla Lazzi, Mitsuoki Kawano, Claudio Rivetti

and Claudia Folli

Fluorescence image processing

Quantification of fluorescence images was performed using *ad hoc* written Matlab scripts. For nucleoid compaction determination the area of the nucleoid was measured as follows: first, the *rgb* DAPI fluorescence image was converted to grayscale and the contrast was enhanced using bottom-hat and top-hat filtering, followed by correction of the image shading distortion using the flat-field correction routine *imflatfield*. Regions of local maximum intensities were identified with the extended-maxima transform of the image (*imextendedmax*). Background artifacts or cell aggregates were manually removed by visual inspection of the images. For each cell, the nucleoid area in pixels was computed using the *regionprops* function.

Percentage of red cells obtained with DAPI/EtBr staining was determined with the following procedure: the *rgb* fluorescence image was imported and the background was removed with the top-hat filter. The resulting *rgb* image was both converted to grayscale and split into channels. The blue channel was subtracted from the grayscale image to filter out blue cells. Conversely, the red channel was subtracted from the grayscale image to filter out red cells. The computed grayscale images were binarized with *imbinarize* using the computed *graythresh* global threshold. 8-connected objects were identified and measured with *bwlabel* with *regionprops* functions. Small background artifacts or large cell aggregates were removed with the following filter: $400 < area < 2000$ & $perimeter < 250$ (pixel units). The remaining objects were counted to obtain the number of either blue or red cells.

Bright-field and fluorescence images were combined using ImageJ. *E. coli* cell were outlined in the bright-field image using the auto local threshold method “Phansalkar”. The image mask, obtained by applying the morphological operations *erosion*, *dilation* and *fill holes* to the binary image, was used to compute the image overlay.

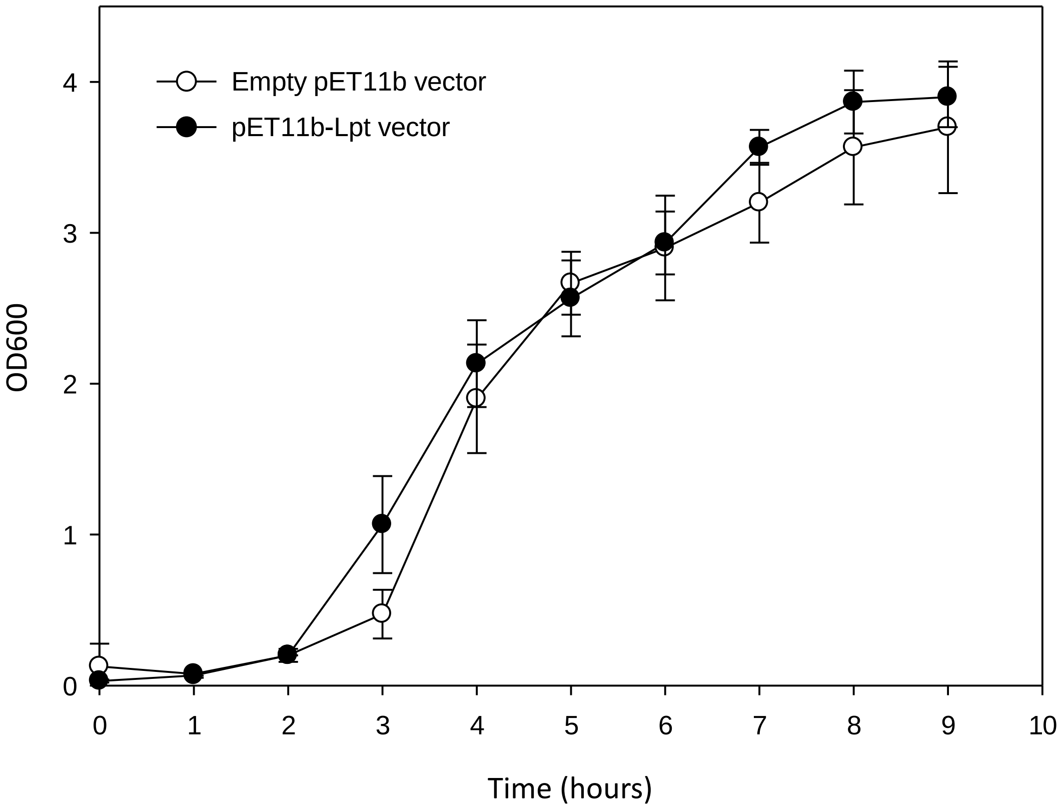

Fig S1. Growth curves of *E. coli* C41(DE3) pLysS strain harbouring an empty pET11b vector (open circles) and a pET11b-Lpt vector (closed circles) in the absence of IPTG. Each data point represents the mean value ±SEM of three independent experiments.

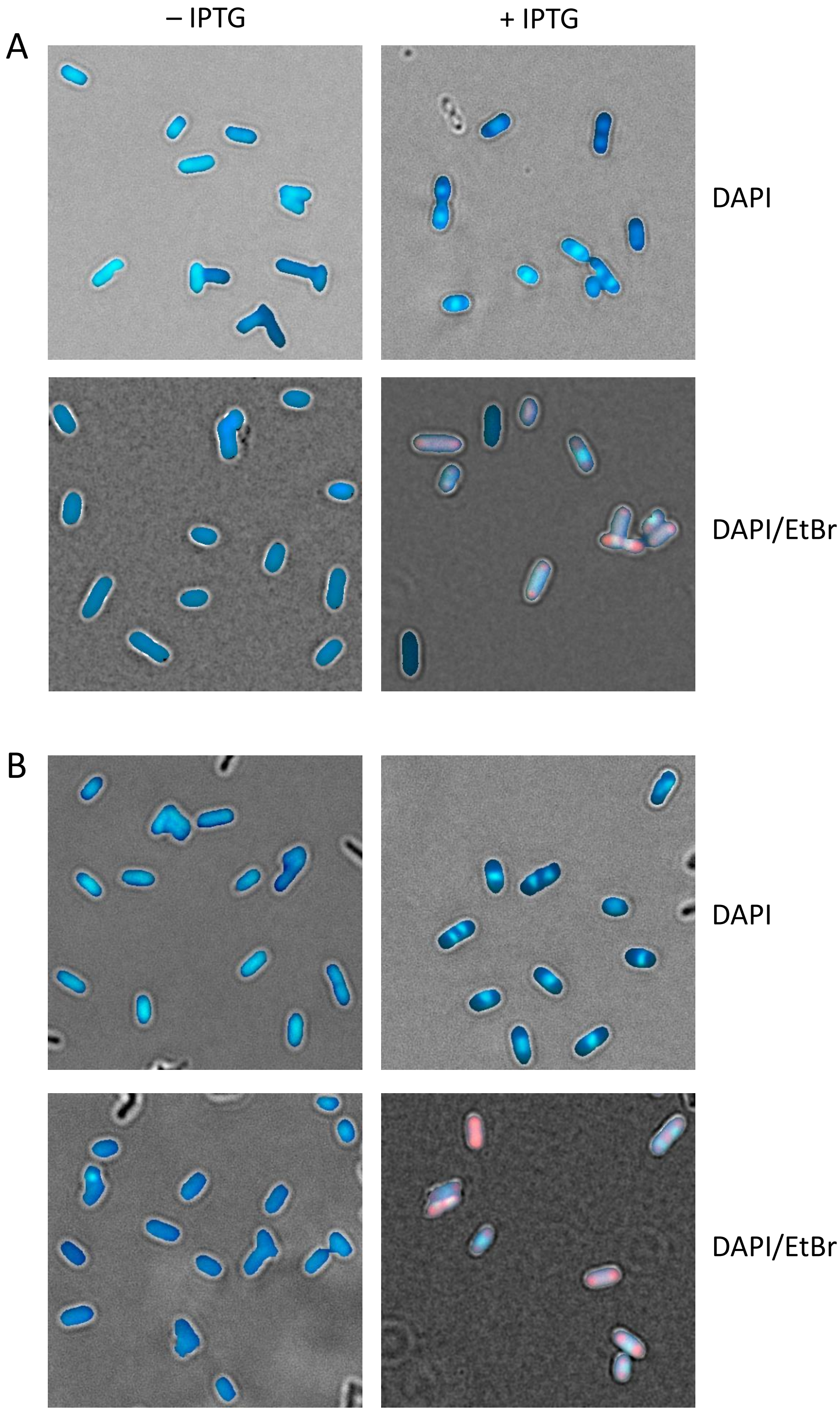

Fig S2. Representative bright-field/fluorescence overlaid images of not induced (left panels) and induced (right panels) *E. coli* cells expressing Lpt P11A (A) and Lpt P11V (B) stained with DAPI (upper panels) or with DAPI/EtBr (lower panels). The quantitative analysis is reported in Fig 4C and D.

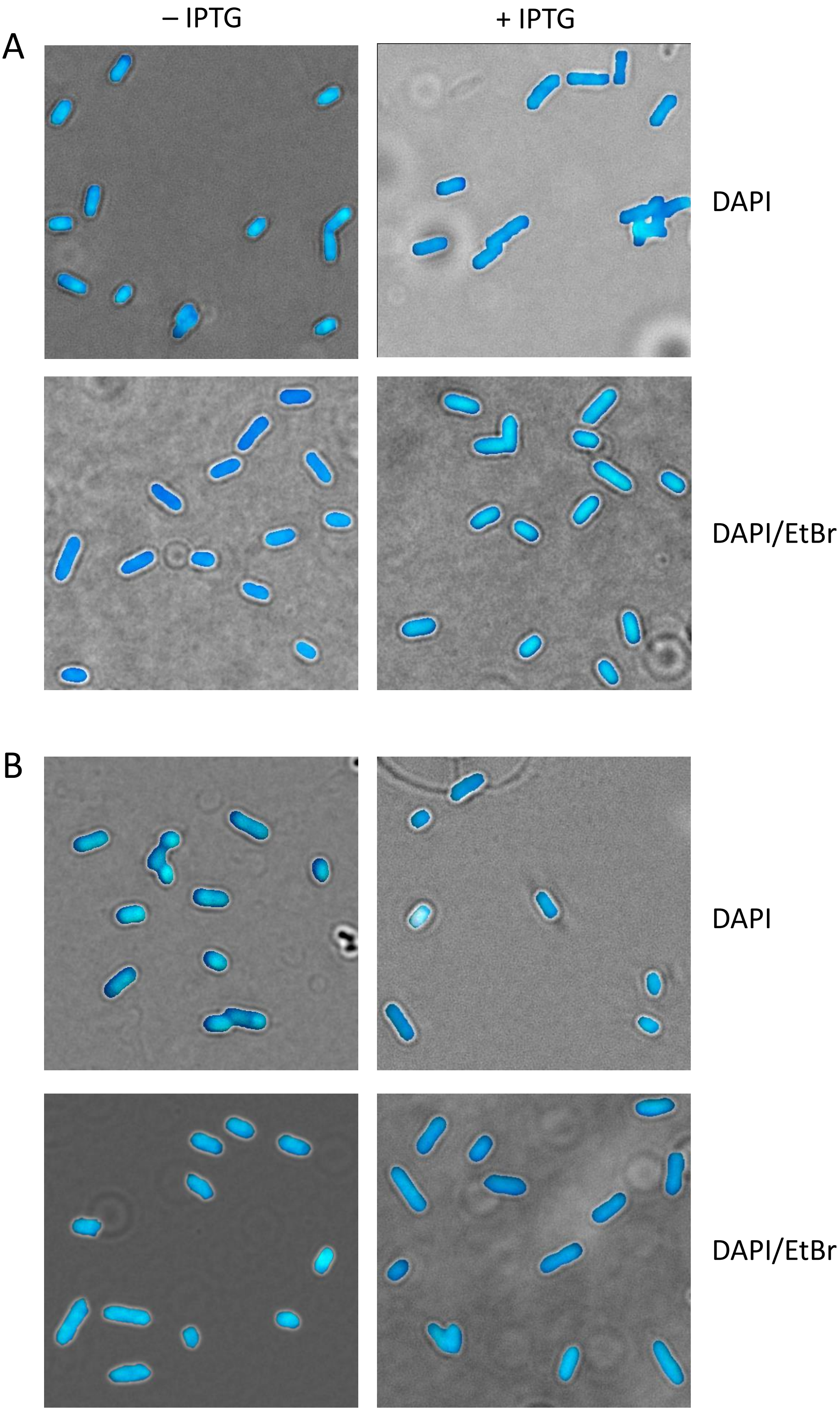

Fig S3. Representative bright-field/fluorescence overlaid images of not induced (left panels) and induced (right panels) *E. coli* cells expressing Lpt P11E (A) and Lpt K22* (B) stained with DAPI (upper panels) or with DAPI/EtBr (lower panels). The quantitative analysis is reported in Fig 4C and D.

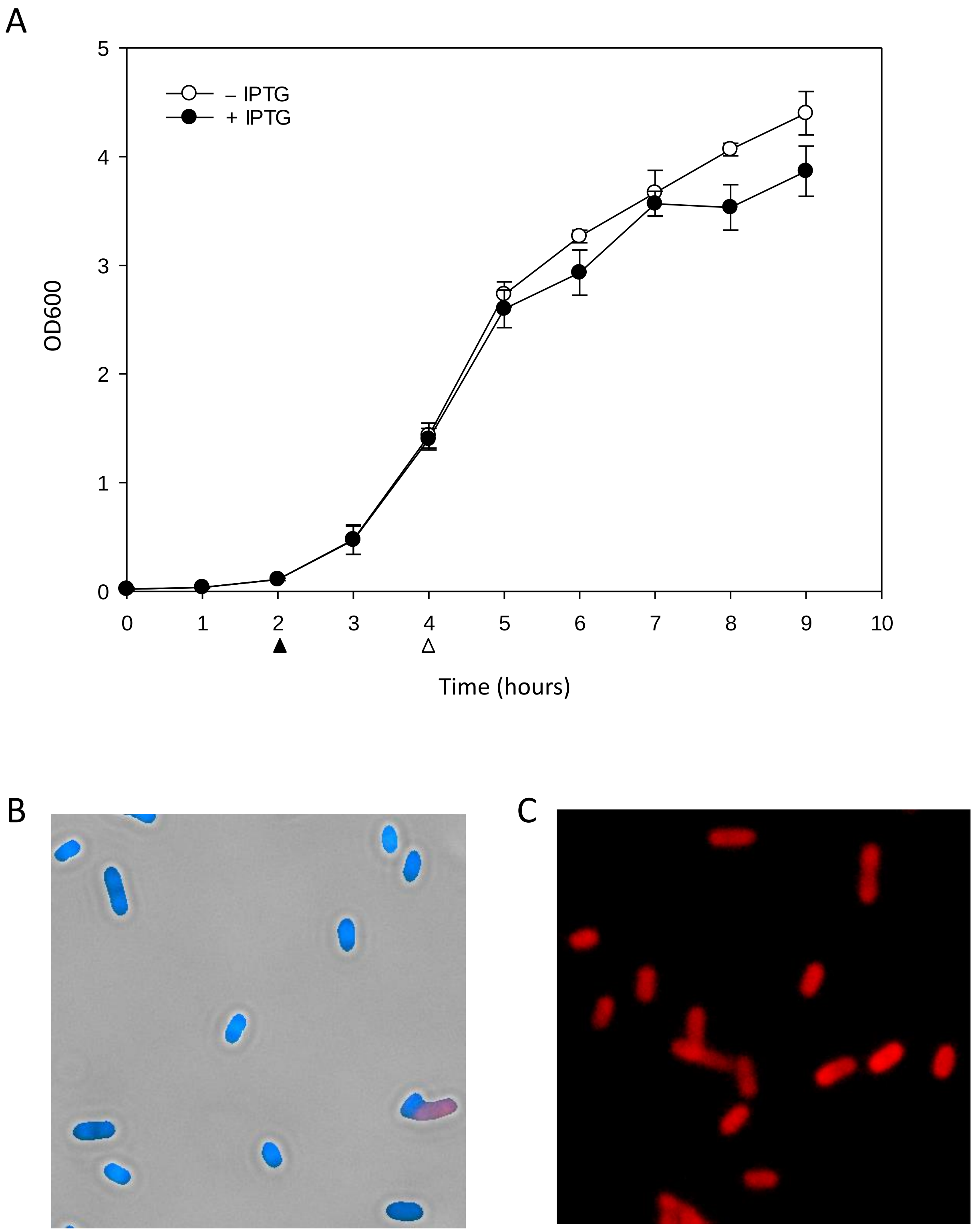

Fig S4. Toxicity and cellular localization of P11E Lpt-mCherry fusion protein. (A) Growth curves of *E. coli* C41(DE3) pLysS strain harbouring the pET11b-LptP11E-mCherry vector in the absence (open circles) and in the presence (closed circles) of IPTG. Each data point represents the mean value ±SEM of three independent experiments. Filled and empty arrows indicate the time points of IPTG addition and cell harvesting for microscopy analysis, respectively. (B) Representative bright-field/fluorescence overlaid image of induced *E. coli* C41(DE3) pLysS expressing P11E Lpt-mCherry fusion protein and stained with DAPI/EtBr. (C) mCherry fluorescence signal of induced *E. coli* C41(DE3) pLysS expressing P11E Lpt-mCherry fusion protein.

A

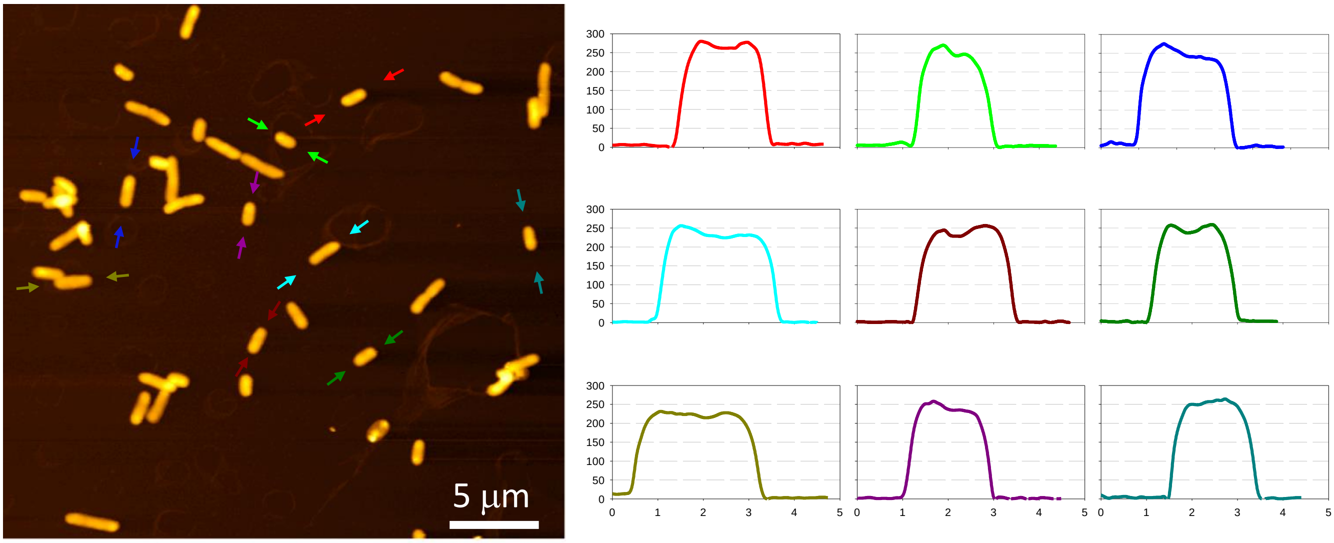

B

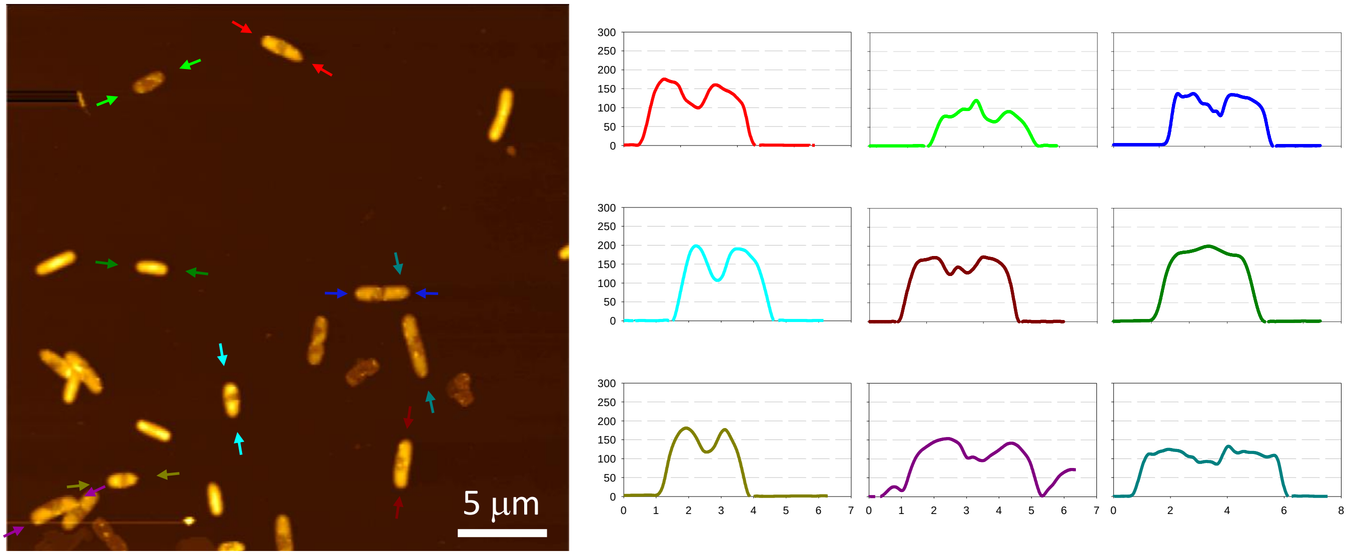

Fig S5. Surface profiles of *E.coli* cells expressing wt Lpt imaged by AFM. Not induced cells (A) and cells induced for two hours (B). Arrows indicate the direction of the profile while the color indicates the corresponding plot shown on the right.

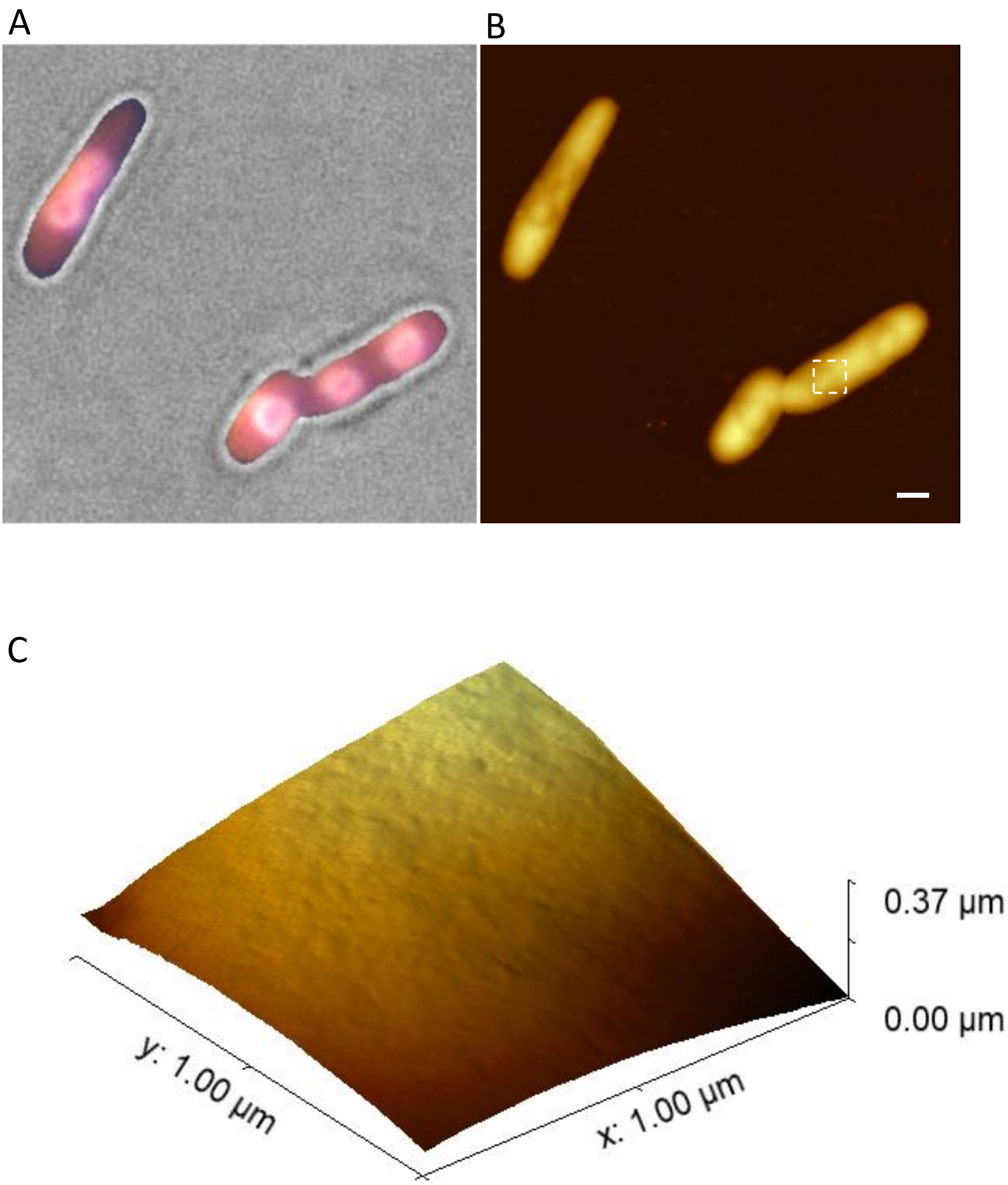

Fig S6. Effect of chloramphenicol on *E. coli* nucleoid morphology and membrane integrity. (A) Representative bright-field/fluorescence overlaid image of *E.coli* cells treated with 200 μg/ml of chloramphenicol for 90 minutes and stained with DAPI/EtBr. (B) AFM image of *E. coli* cells shown in A. Bar equal to 1 μm. (C) 3D view of cell surface details.

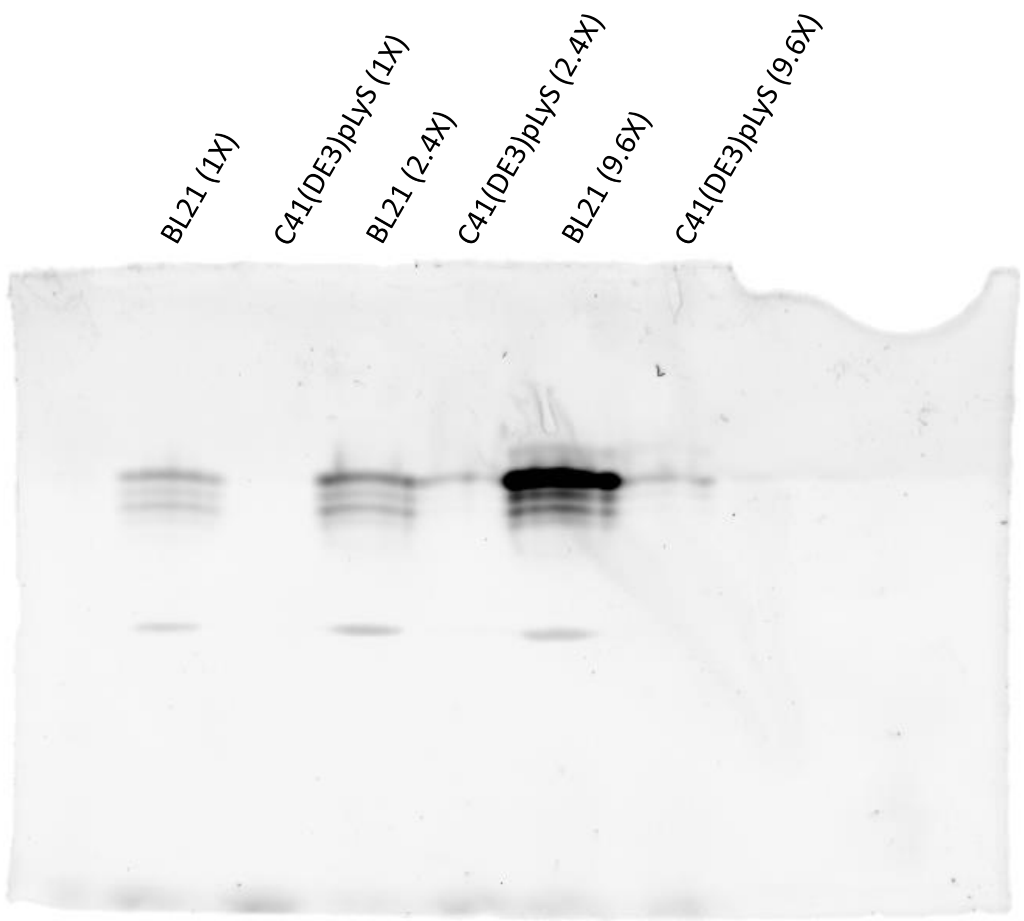

Fig S7. Expression levels of mCherry in BL21 and C41(DE3)pLyS *E. coli* cells transformed with the pET11b-mCherry plasmid. Fluorescence image of 15% SDS PAGE under mild denaturing conditions of bacteria harvested after two hours of IPTG induction and resuspended in 1% SDS. 1X represents the amount of cells present in 5 µl of a bacterial suspension having an OD600 of 3.3. Bands represent different partially unfolded states of mCherry. The total fluorescence signal per lane was recorded with a Bio-Rad ChemiDoc imager using the Alexa 546 mode.

Supplementary Table S1. Oligonucleotides used in this work. Modified codons are underlined.

Primer	Sequence	Application
Lpt-plus	CATATGAATTCATTCGATAAAGCGA	wt Lpt cloning
Lpt-minus	GGATCCAAGCCATCATCTCCG	wt Lpt cloning
LptP11A-plus	GCGATCATCGCG<u>GCG</u>CTGCTTGTCGG	mutagenesis
LptP11A-minus	CCGACAAGCAG<u>CGC</u>CGCGATGATCGC	mutagenesis
LptP11V-plus	GCGATCATCGCG<u>GTG</u>CTGCTTGTCGGTGTG	mutagenesis
LptP11V-minus	CACACCGACAAGCAG<u>CAC</u>CGCGATGATCGC	mutagenesis
LptP11E-plus	GCGATCATCGCG<u>GAG</u>CTGCTTGTCGGTGTG	mutagenesis
LptP11E-minus	CACACCGACAAGCAG<u>CTC</u>CGCGATGATCGC	mutagenesis
LptK22stop-plus	GTGTTTCTACTTTTGTTG<u>TAA</u>TACGCGCTGGATAACCAC	mutagenesis
LptK22stop-minus	GTGGTTATCCAGCGCGT<u>ATT</u>ACAACAAAAGTAGAAACAC	mutagenesis
LptP11EmCherry-plus	GGCGATCATTGCG<u>GAG</u>CTGCTGGTTGGTGTG	mutagenesis
LptP11EmCherry-minus	CACACCAACCAGCAG<u>CTC</u>CGCAATGATCGCC	mutagenesis

Supplementary Table S2. DNA plasmids used in this work.

Plasmid	Application
pGEM-Lpt	Cloning of Lpt coding sequence
pET11b-Lpt	Expression of wt Lpt
pET11b-LptP11A	Expression of P11A Lpt mutant
pET11b-LptP11V	Expression of P11V Lpt mutant
pET11b-LptP11E	Expression of P11E Lpt mutant
pET11b-LptK22*	Expression of truncated Lpt
pET11b-LptmCherry	Expression of Lpt-mCherry fusion protein
pET11b-mCherry	Expression of mCherry
pET11b-LptP11E-mCherry	Expression of P11E Lpt-mCherry fusion protein
